# The Beneficial Effects of n-3 Polyunsaturated Fatty Acids on Diet Induced Obesity and Impaired Glucose Control Do Not Require Gpr120

**DOI:** 10.1371/journal.pone.0114942

**Published:** 2014-12-26

**Authors:** Mikael Bjursell, Xiufeng Xu, Therése Admyre, Gerhard Böttcher, Sofia Lundin, Ralf Nilsson, Virginia M. Stone, Noel G. Morgan, Yan Y. Lam, Leonard H. Storlien, Daniel Lindén, David M. Smith, Mohammad Bohlooly-Y, Jan Oscarsson

**Affiliations:** 1 AstraZeneca R&D, Mölndal, Sweden; 2 University of Exeter Medical School, RILD Building, Barrack Road, Exeter, EX2 5DW, United Kingdom; 3 University of Sydney, Sydney, Australia; 4 Pennington Biomedical Research Centre, Baton Rouge, Louisiana, United States of America; Northeast Ohio Medical University, United States of America

## Abstract

GPR120 (Ffar4) has been postulated to represent an important receptor mediating the improved metabolic profile seen upon ingestion of a diet enriched in polyunsaturated fatty acids (PUFAs). GPR120 is highly expressed in the digestive system, adipose tissue, lung and macrophages and also present in the endocrine pancreas. A new *Gpr120* deficient mouse model on pure C57bl/6N background was developed to investigate the importance of the receptor for long-term feeding with a diet enriched with fish oil. Male *Gpr120* deficient mice were fed two different high fat diets (HFDs) for 18 weeks. The diets contained lipids that were mainly saturated (SAT) or mainly n-3 polyunsaturated fatty acids (PUFA). Body composition, as well as glucose, lipid and energy metabolism, was studied. As expected, wild type mice fed the PUFA HFD gained less body weight and had lower body fat mass, hepatic lipid levels, plasma cholesterol and insulin levels and better glucose tolerance as compared to those fed the SAT HFD. *Gpr120* deficient mice showed a similar improvement on the PUFA HFD as was observed for wild type mice. If anything, the Gpr120 deficient mice responded better to the PUFA HFD as compared to wild type mice with respect to liver fat content, plasma glucose levels and islet morphology. *Gpr120* deficient animals were found to have similar energy, glucose and lipid metabolism when fed HFD PUFA compared to wild type mice. Therefore, GPR120 appears to be dispensable for the improved metabolic profile associated with intake of a diet enriched in n-3 PUFA fatty acids.

## Introduction

GPR120 is a G-protein coupled receptor that is highly expressed in the human and rodent digestive system, notably, though not exclusively, in enteroendocrine L-cells [1]. In the intestine, GPR120 mediates free fatty acid (FFA) stimulated release of glucagon-like peptide 1 (GLP1) that increases glucose stimulated insulin secretion (GSIS), enhances β-cell mass and reduces gastric emptying and appetite [2]. Germ free mice given access to intralipid emulsions display significantly reduced intestinal expression of GPR120, indicating that expression of this receptor is dependent on the intestinal lipid content and microbiota [3]. In addition to its role in the intestine, GPR120 is also expressed in adipose tissue, lung, pro-inflammatory macrophages and islets of Langerhans [2], [4]–[6]. GPR120 was recently shown to be expressed in the delta-cells of the islets of Langerhans mediating a negative effect on glucose stimulated somatostatin secretion [7] as well as in alpha-cells mediating the fatty acid induced secretion of glucagon [8].

Long chain fatty acids (LCFAs) are preferred ligands for GPR120 [2], [9], [10] [5], [11]. The most potent GPR120 ligands are n-3 polyunsaturated fatty acids (PUFAs), such as α-linolenic acid (ALA), eicosapentaenoic acid (EPA) and docosahexaenoic acid (DHA) [5], [11]. However, also n-6 PUFA and saturated fatty acids are able to activate the receptor [2], [8].

Mice deficient in *Gpr120* have been developed and studied in relation to diet-induced obesity and insulin resistance [5], [6] [8]. Oh *et. al.* performed studies on *Gpr120* deficient mice having a mixed 129Sv/C57BL/6 genetic background and exon 2 replaced by a neomycin selection marker. The *Gpr120* deficient mice, showed impaired glucose tolerance, increased insulin secretion, as well as hepatic and skeletal muscle insulin resistance on normal chow diet (containing exogenous ω-3 lipids) despite having unaltered body weights [5]. Both WT and *Gpr120* deficient mice were similarly susceptible to the development of insulin resistance when fed a HFD without n-3 PUFA supplementation [5]. However, unlike their wild type counterparts, *Gpr120* deficient mice did not display improvements in insulin sensitivity and hepatic lipid content when fed a high fat diet (HFD) supplemented with n-3 PUFA [5]. The findings by Oh and co-workers suggested that GPR120 is necessary for the beneficial effects of n-3 PUFA on glucose control and insulin action and, as such, supported earlier observations on the effects of n-3 PUFA [12], [13] and reviewed in [14], [15]. It was recently shown that *Gpr120* deficient mice from the same provider as used by Oh *et. al.* [5], and back-crossed to C57bl/6, had higher body weight gain on chow diet [8]. On HFD diet, the Gpr120 deficient animals were heavier than controls at younger age, but the difference disappeared at 4 months of age. Moreover, they showed an increased glucagon secretion and sensitivity that could help to explain the observation of higher plasma glucose levels and impaired glucose tolerance in the *Gpr120* deficient mice. It was concluded that the *Gpr120* deficient mice were hyperglycaemic and glucose intolerant because of an hyperactive counter-regulatory response rather than insulin resistance [8]. Another study have reported the phenotype of a different *Gpr120* deficient mouse line generated on a mixed 129Sv/C57BL/6 genetic background with exon 1 in the *Gpr120* locus replaced by a neomycin resistance selection marker [6]. These *Gpr120* deficient mice were not different from wild-type controls with respect to body weight, fasting plasma glucose or insulin levels when fed a chow diet. However, when fed a 60% HFD with similar amounts of saturated and mono-unsaturated lipids and low n-3 fatty acids, the *Gpr120* deficient mice displayed higher body weight, body fat mass and liver fat as well as elevated fasting plasma glucose and insulin levels as compared to the control mice [6]. In summary, the combined results from published studies do not give a clear picture of the role of GPR120 for the effects of n-3PUFA in relation to saturated long-chain fatty acids.

In the present study, a new independent *Gpr120* deficient mouse line was developed on a pure C57bl/6N genetic background with exon 1 disrupted by an ATG-*LacZ* gene fusion and without carrying any antibiotic selection marker. These mice have been used recently to investigate the distribution of the receptor, especially in the islets of Langerhans, and importance of GPR120 for the regulation of somatostatin and insulin secretion [7]. The mice in the present study were fed either a HFD based on lard and palm oil in which most lipids are saturated fatty acids (SAT HFD) or alternatively they were fed a HFD based on Menhaden oil, which contains predominantly n-3 polyunsaturated fatty acids (PUFA HFD). The primary aim of the study was to investigate the effects of the PUFA diet as compared to the saturated fat diet in wild-type (WT) mice and in *Gpr120* deficient mice.

## Material and Methods

### Generation of Gpr120 null mice

All experiments were approved by Gothenburg Ethics Committee for Experimental Animals.

**Figure 1:**
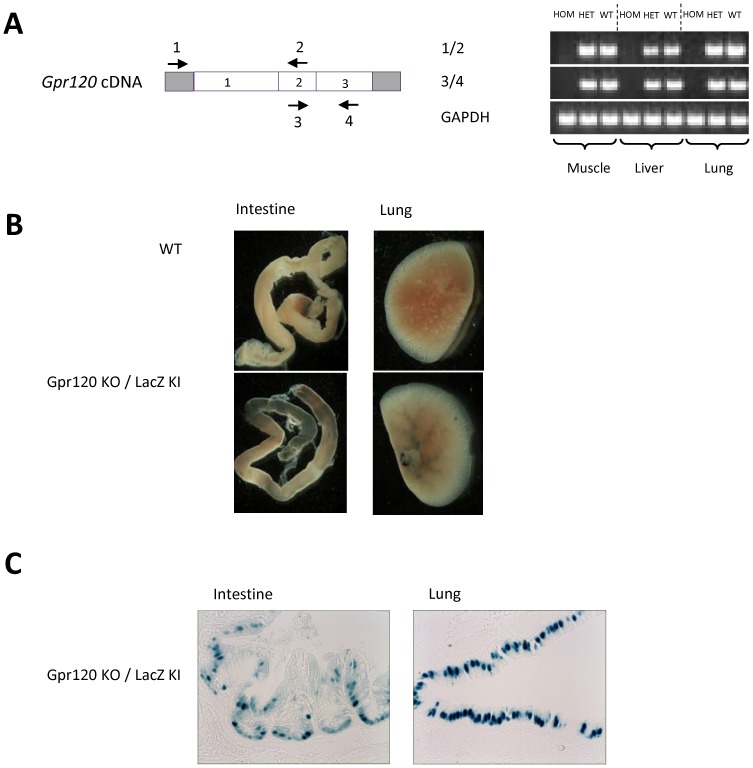
Validation of the genetic model. A; *Gpr120* transcript and RT-PCR analysis. Tissues were dissected from wild type, *Gpr120* heterozygous and *Gpr120* homozygous littermate mice. Arrows indicate oligos used for RT-PCR. B; Representative pictures of *LacZ* expression by X-gal induced staining in lung and intestine dissected from wild type and *Gpr120* KO mice. C; Tissue slides of intestine and lungs derived from *Gpr120* KO mice stained with X-gal. Magnification was set to 40x.

The targeting strategy of the mouse *Gpr120* gene is described under **S1 Supplementary experimental procedures** and illustrated in **S1A Fig.** In short, a 0.567 kb fragment of the coding sequence (CDS) within exon 1 was replaced in frame by a nuclear βGal (*nβGal*) expression cassette and a loxP floxed PGKneo selection marker gene. This resulted in the deletion of transmembrane domains 1–4 of the GPR120 protein and allowed the expression of *nβGal* to be driven by the endogenous *Gpr120* promoter. The mice were genotyped by PCR using primers amplifying a wild type allele (0.299 kb fragment) and the null allele (0.580 kb fragment), forward: 5'-GCTTTCATATGGGGTTACTCG-3'; reverse: 5'-ACTTGGCACTGTGGGTAAACT-3'; 732, forward: 5'-TGAAGGCTCTTTACTATTGCT-3'. Tissue samples from lung, liver and skeletal muscle were dissected from 8 week old wild type, *Gpr120* heterozygous and *Gpr120* homozygous littermates. Total RNA was extracted with TRIZol Reagent (Invitrogen) according to the manufacturer's protocol. Reverse transcription was performed with SuperScript First-Strand (Invitrogen) followed by PCR using primers located in 5′ UTR of exon 1 of *Gpr120* and within downstream intact exons, forward: 5'-ATGAGCGCTCTCTCAGACAGC-3'; reverse: 5'-GCCAATCCAATGTGCAAATCG-3'; forward: 5'-ATTGGCCCAACCGCATAGGAG-3' and reverse: 5'-TCATTTCGCCTGACAGACGTA-3' (**Fig. 1A**). Tissue X-gal staining experiment was performed as described previously [16] but the tissues were stained at 37°C over night (**Fig. 1B**).

### Animal experiments

The *Gpr120* heterozygous mouse colony was expanded by breeding to C57Bl/6N mice (Charles River) and heterozygous intercross was performed to produce experimental (*Gpr120* KO) and wild type (WT) littermate control cohorts, having a pure C57bl/6N genetic background. Male *Gpr120* KO and WT littermates were housed individually in a temperature controlled room (22°C) with a 12 hour light-dark cycle. They had access to a normal chow diet (R36, Lactamin AB, Stockholm, Sweden) and water *ad libitum*. The R36 chow diet contained (weight %): 3.5% cellulose, (energy %): 22.9% protein, 67.1% carbohydrate and 9.6% fat. The main sources of proteins were from soy, grain and potatoes. Carbohydrate source was mainly grains and the main fat source was soy beans. Fatty acid composition of the R36 chow diet is the following; C16:0∼18%; C18:1 n9∼16%; C18:2 n6∼53%; C18:3 n3∼5% (remaining n3 FAs <0.1%). The energy density of R36 is 3.08 kcal/g. During high fat diet (HFD) feeding, two different HFD were used (Research Diets Inc., New Brunswick, USA). Both diets had the following energy source composition (energy %): proteins 20%; carbohydrates 35%, lipids 45% and an energy density of 4.73 kcal/g. The lipids from the polyunsaturated (PUFA) HFD were derived from Menhaden oil and contained 29% saturated fat, 24% monounsaturated fat and 47% polyunsaturated fat, resulting in 11 g/kg n-6 lipids, 75 g/kg n-3 lipids and an n-6/n-3 ratio of 0.14. The lipids from the saturated (SAT) HFD were derived from lard (50%) and palm oil (50%) and contained 42% saturated fat, 45% monounsaturated fat and 13% polyunsaturated fat, resulting in 27 g/kg n-6 lipids, 2 g/kg n-3 lipids and an n-6/n-3 ratio of 15.33. Detailed information on the diets is presented in **S1 Table**.

The mice were initially fed the normal R36 chow diet. At 13 weeks of age, they were subdivided into two groups, and 1 cohort of *Gpr120* KO mice (*n = 7*) and 1 cohort of WT mice (*n = 8*) were switched to the SAT HFD while a second cohort of *Gpr120* KO mice (*n = 7*) and WT mice (*n = 8*) were switched to the PUFA HFD. The HFD was provided to all animals over an 18 week period. Separate groups of WT (n = 8) and *Gpr120* KO (n = 8) mice were fed R36 chow diet for 16 weeks before their body composition was analysed.

All mice were terminated at 31 weeks of age, 18 weeks after the introduction of their respective HFDs. The mice were fasted for 3 hours, then anaesthetised by Isoflurane inhalation and blood was collected in EDTA-coated tubes by cardiac puncture. Blood plasma was separated by centrifugation (2500 rpm (664 g), 10 min. 4°C) and snap frozen in liquid nitrogen. Liver tissue samples were taken for hepatic triglyceride content analysis [17]. Tissue samples of liver, epididymal WAT and pancreas were processed for histological analysis by immersion fixation in 4% buffered formaldehyde solution for 24–48 hours, dehydrated in graded series of alcohol and embedded in paraffin, sectioned and stained.

### Body weight, indirect calorimetry, locomotor activity, food intake, body temperature and composition

Body weights of the *Gpr120* KO and WT mice were recorded on a weekly basis from 4 weeks of age up to 23 weeks of age and then finally at 31 weeks of age. Body length (nose to base of the tail) was assessed at 23 weeks of age. Assessment of indirect calorimetry, water consumption and locomotor activity was performed at 22–23 weeks of age (10 weeks after HFD introduction) in a CLAMS system (Columbus Instruments, Columbus, USA) at thermoneutral temperature (set for WT mice to be 29.5°C) as previously described [18]. The mice were placed in the CLAMS calorimeter chambers with *ad libitum* access to diet and water for 72 hours. Energy intake was analysed over 48 hours in food deprived mice (12 hours) as previously described [19] with a minor modification: no initial incubation (80°C for 1 hour) of the cages was done. Total faeces produced over the measurement periods were collected and the energy content of the faeces was determined with a bomb calorimeter (C 5000, IKA^®^ Werke GmbH & Co. KG, Germany). Rectal core body temperatures were recorded in conscious non-anaesthetised mice during day time (10.00–11.00 am) using a rectal probe [18]. Body composition was assessed by dual energy X-ray absorptiometry (DEXA, GE Lunar, Madison, USA) in Isoflurane anaesthetised mice as previously described [18].

### Oral glucose tolerance test (OGTT)

OGTT was performed 14 weeks after respective HFD introduction as previously described [20]. Fasting blood glucose level times fasting insulin level was calculated [fasting blood glucose (mM) x fasting blood insulin (ng/ml)] as an index of insulin resistance.

### Plasma analysis

Plasma levels of cholesterol, triglyceride, leptin, adiponectin, alanine aminotransferase (ALT) and albumin were determined as previously described [17]. Plasma levels of fructosamine were measured using an enzymatic colorimetric method (Kit No FR2992, Randox Laboratories Ltd, UK) and assays were performed on an ABX Pentra 400 (Horiba ABX, France). Total bilirubin was measured using a colorimetric method (Kit No 11552414 216; BIL-T, Roche Diagnostics GmbH, Germany). Plasma lipids were extracted as described previously [21] and explained in detail under S1 Supplementary experimental procedures. Individual fatty acids, including, C14:0, C16:0, C16:1*n*-9, C18:0, C18:1*n*-9, C18:2*n*-6, C18:3*n*-3 (ALA), C20:4*n*-6, C20:5*n*-3 (EPA), C22:6*n*-3 (DHA) were quantified by calculating area response versus the internal standard.

### Histology

Epididymal WAT macrophage staining and semi quantitative assessment were performed on histological sections as previously described using an anti-Mac2/galectin3 antibody [17]. Adipocytes were also double stained with Perilipin and Mac2/gelectin3 antibodies, details are outlined in **S1 Supplementary experimental procedures**. Histopathological examination and evaluation of liver tissue samples was performed on hematoxylin-eosin (H&E) stained sections and degree of steatosis and inflammation was scored on a semi quantitative 5 grade scale. Serial sections of paraffin embedded pancreases were employed for immunostaining and were prepared from WT mice fed chow (*n = 3 separate group*), SAT HFD or PUFA HFD and from *Gpr120* KO mice fed chow (*n = 3 separate group*), SAT HFD or PUFA HFD. Sections were stained with anti-insulin (Dako Cytomation, Ely, UK) and anti-Mac2 (Cederlane Labs, Ontario, Canada) antibodies (DAKO, Ely, UK) using standard immunoperoxidase technique (see S1 Supplementary experimental procedures). Slides were examined by light microscopy and quantitative analysis carried out using randomly selected islets from each section. The number of Mac2/galectin3 positive cell profiles (indicating the number of macrophages) present within the islet profile or in the peri-islet area was recorded. The area of each islet was measured using ImageJ software.

### Statistical analysis

All values are given as group means ± SEM. Statistical analyses was performed using 1-way ANOVA and if significant (p<0.05) followed by pair-wise comparison using Student's t-test between the two HFD groups in WT and *Gpr120* KO mice, respectively. The other 4 possible comparisons were not tested. Statistical calculations of parameters measured over time were done by a 2-way ANOVA using time and diet as factors or alternatively calculating AUC for each observation and then applying 1-way ANOVA. Data was log normalized when appropriate. p<0.05 between the groups was considered to be statistically significant differences.

## Results

*Gpr120* null animals were generated by targeted deletion of a part of exon 1 in the *Gpr120* locus (**S1A Fig.**). *Gpr120* deficiency was confirmed by RT-PCR analyses, designed to amplify fragments both within and outside the deleted DNA sequence, using RNA derived from skeletal muscle, liver and lung tissue from wild type, heterozygous and homozygous *Gpr120* KO mice. As expected, no expression of *Gpr120* was observed in the homozygous *Gpr120* KO mice (**Fig. 1A**). The construct design was validated by *LacZ* expression in which blue staining was observed in tissue sections where GPR120 is known to be present upon incubation with X-gal. Staining was observed in the lung and the intestine of *Gpr120* deficient mice but was absent from all tissues in WT mice (**Fig. 1B**). Slides from intestine and lungs clearly show positive staining in enteroendocrine cells and goblet cells, respectively (**Fig. 1C**). Intercrossing of male and female mice heterozygous for the *Gpr120* mutation resulted in offspring of normal litter sizes. Among the male offspring; 26% were homozygous for the deletion, 48% were heterozygous and 26% were wild type.

### Body weight and body composition

**Figure 2:**
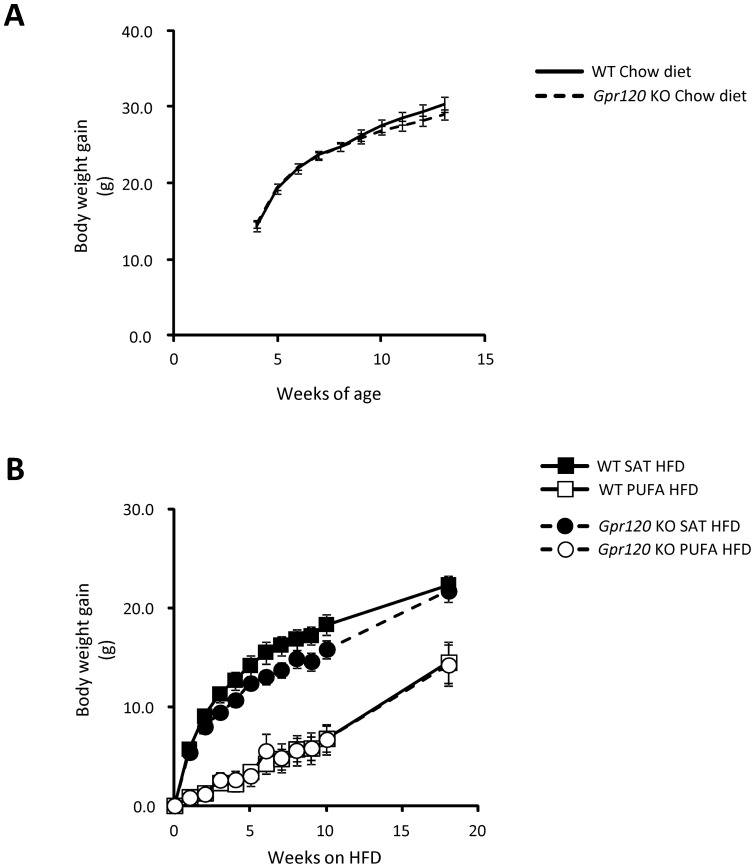
Body weight gain. A; Body weight gain from four to thirteen week of age during chow diet feeding in WT mice (n = 16, solid line) and *Gpr120* KO (n = 14, dashed line). B; Body weight gain over an 18 week period of feeding of HFDs in WT fed SAT HFD (n = 8, filled square) and PUFA HFD (n = 8, open square) and in *Gpr120* KO mice fed SAT HFD (n = 7, dashed line, filled circle) and PUFA HFD (n = 7, dashed line, open circle). Statistical analysis was done by 1-way ANOVA for each time point followed by pair wise comparisons by Student's t-test using a pooled estimate of variability from the ANOVA. Body weight was significantly lower in the PUFA HFD fed mice at all time points assessed compared to mice fed SAT HFD.

No significant differences in body weight gain were observed between Gpr120 KO *(n = 14)* and WT *(n = 16)* mice on chow diet at any time point up to 13 weeks of age (**Fig. 2A**). Moreover, body composition was assessed by DEXA in a separate cohort of chow fed *Gpr120* KO and WT mice at 16 weeks of age. At that time, there was no significant difference in absolute and relative measures of body lean mass, body fat mass, bone mineral content (BMC) or bone mineral density (BMD) (data not shown). The mice in this cohort were also studied with respect to assessment of body weight gain, indirect calorimetry, ECG and a number of behavioural assessments [18] over a 48 week period. No significant differences were observed in any of these assessments between chow fed WT and *Gpr120* KO mice (data not shown).

After switching to SAT HFD or PUFA HFD at 13 weeks of age, no significant differences in body weight gain were observed between the WT and *Gpr120* KO mice (**Fig. 2B**). However, PUFA HFD feeding resulted in lower body weight gain in both genotypes. At study termination after 18 weeks on HFDs, the mice fed SAT HFD were more than 20% heavier than the mice on PUFA HFD (*p<0.05*). Body length did not differ significantly between any of the groups (data not shown).

**Figure 3:**
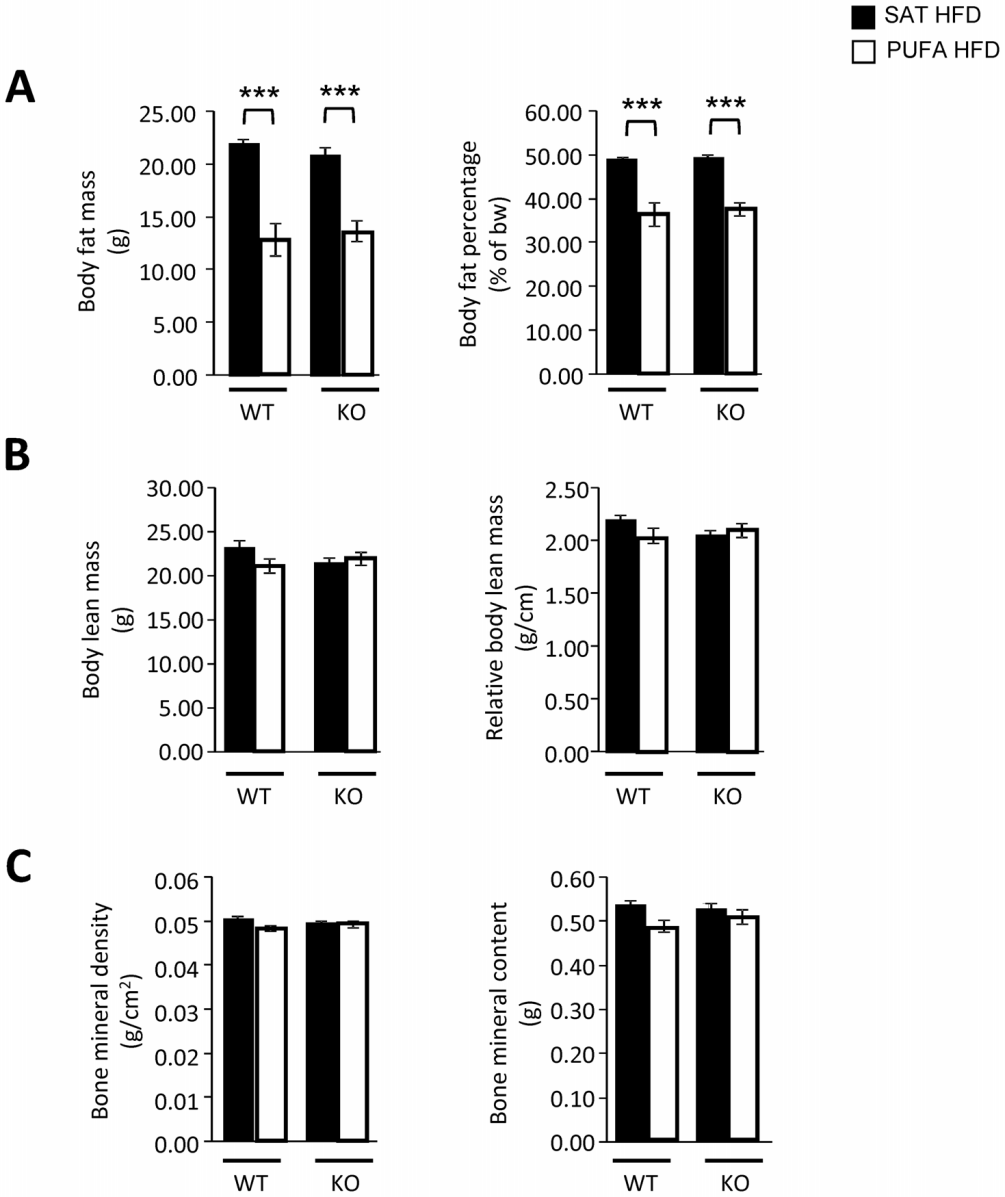
Body composition analyses. Body composition was assessed at 23 weeks of age after 11 weeks of HFD. A; body fat mass, B; body lean mass and C; body bone mineral density (BMD) and content (BMC) in WT mice fed SAT HFD (n = 8, filled bars) and PUFA HFD (n = 8, open bars) and in *Gpr120* KO mice fed SAT HFD (n = 7, filled bars) and PUFA HFD (n = 7, open bars). Statistical analysis was done by 1-way ANOVA followed by two comparisons (SAT HFD *vs*. PUFA HFD) using Student's t-test, *** p<0.001.

Assessment of body composition was performed after 11 weeks on HFD (23 weeks of age). Both WT and *Gpr120* KO mice fed PUFA HFD had significantly lower absolute and relative (% of body weight) body fat mass compared to WT mice fed the SAT HFD (**Fig. 3**). Lean body mass was not significantly different between animals on PUFA HFD as compared to SAT HFD in any of the genotypes. Also, no significant effects on bone mineral density (BMD) or bone mineral content (BMC) were observed between mice fed PUFA *vs*. SAT HFD regardless of genotype. (**Fig. 3**).

### Energy intake, energy expenditure, locomotor activity and core body temperature

The major difference in body composition between mice on PUFA HFD and SAT HFD was body fat mass. Since energy expenditure in adipose tissue is generally relatively low, energy intake and energy expenditure are presented per individual mouse as well as related to lean body mass. Energy intake per mouse (kcal/day) was significantly higher when *Gpr120* KO mice were fed PUFA HFD compared to SAT HFD. The same trend was also seen in WT mice on PUFA HFD as compared to SAT HFD (**Table 1**). Energy intake related to lean body mass was significantly higher in both WT and *Gpr120* KO mice on PUFA HFD as compared to SAT HFD. Interestingly, also the faecal energy content was increased when the mice were fed PUFA HFD compared to SAT HFD, but the difference was statistically significant in *Gpr120* KO mice only. When taking into account the faecal energy loss, relative energy uptake was significantly higher in PUFA fed WT and *Gpr120* KO mice expressed as energy intake per lean body mass. Also relative water intake was higher when the mice were fed PUFA HFD compared to SAT HFD (**Table 1**).

**Table 1:** Energy intake and faecal energy content.

*Parameter\Genotype*	WT (n = 8) SAT HFD	WT (n = 8) PUFA HFD	*Gpr120* KO (n = 7) SAT HFD	*Gpr120* KO (n = 7) PUFA HFD	1-way ANOVA
Energy intake (kcal/day)	15.31±1.03	17.56±0.88	14.93±0.98	18.03±0.87*	p<0.05
Rel. energy intake (kcal/day/g LBM)	0.66±0.04	0.84±0.05*	0.70±0.04	0.82±0.04*	p<0.05
Faecal energy content (kcal/day)	1.07±0.09	1.38±0.14	1.14±0.12	1.46±0.08*	p<0.05
Energy uptake (kcal/day)	14.24±0.95	16.18±0.76	13.79±0.88	16.57±0.80	NS
Rel. energy uptake (kcal/day/g LBM)	0.61±0.04	0.78±0.05*	0.64±0.04	0.75±0.04*	p<0.05
Water intake (ml/day)	2.28±0.19	2.69±0.14	2.19±0.18	3.12±0.39*	p<0.05
Rel. water intake (ml/day/g LBM)	0.098±0.007	0.129±0.007**	0.104±0.008	0.142±0.020	p<0.05

Values are presented as group mean ± SEM. Rel.  =  relative. LBM  =  lean body mass. Statistical analysis performed by 1-way ANOVA followed by Students T-test comparing SAT HFD *vs*. PUFA HFD. Star indicates significant difference between mice fed SAT HFD vs. WT fed PUFA HFD. * p<0.05; ** p<0.01.

**Figure 4:**
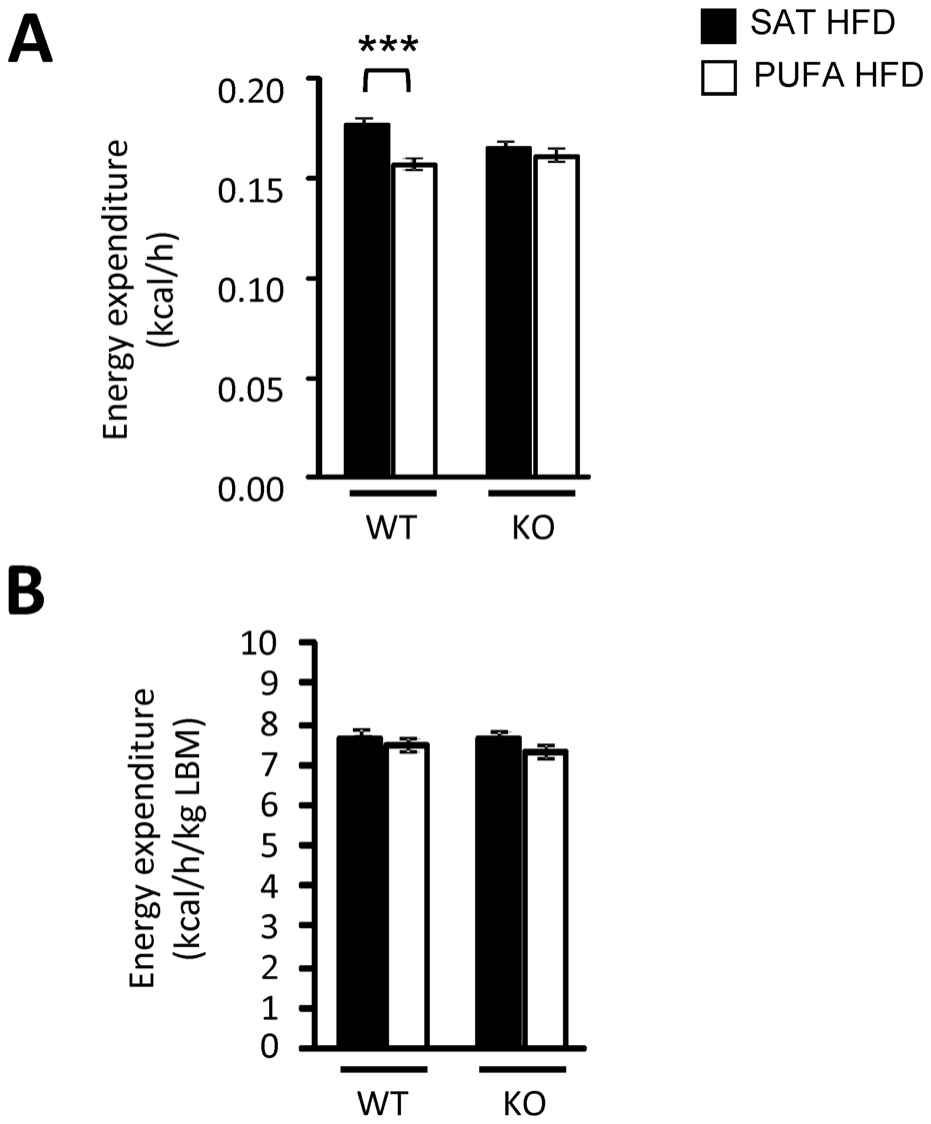
Indirect calorimetry assessment. A; Energy expenditure given in kilocalories per hour per mouse (kcal/h) and B; energy expenditure relative to lean body mass (LBM). The groups are WT fed SAT HFD (n = 8) and PUFA HFD (n = 8) as well as *Gpr120* KO mice fed SAT HFD (n = 7) and PUFA HFD (n = 7). Mean values for energy expenditure over 72 h was calculated for each individual mouse and the graphs show mean values for the treatment groups. Statistical analysis was performed using 1-way ANOVA followed by Student's t-test comparing SAT HFD and PUFA HFD in each genotype, * p<0.05.

Mean values for energy expenditure over 72 h was calculated for each individual mouse and presented as mean values for the treatment groups (**Fig.4**) and values for each 2 h time point during the 72 h period in the CLAMS system are presented in **Fig. S2**. Energy expenditure expressed per mouse was lower in WT mice on PUFA HFD as compared to WT mice on SAT HFD, while there was no significant difference between the groups of *Gpr120* KO mice. However, there was no significant difference in energy expenditure relative to lean body mass between mice given PUFA HFD and mice given SAT HFD, neither in WT nor in *Gpr120* KO animals. No significant difference was observed in respiratory exchange ratio (RER) between mice fed PUFA HFD and SAT HFD, regardless of genotype (data not shown). Neither locomotor activity nor core body temperature was significantly influenced by the diets in WT and *Gpr120* KO mice (data not shown).

### Glucose homeostasis

**Figure 5:**
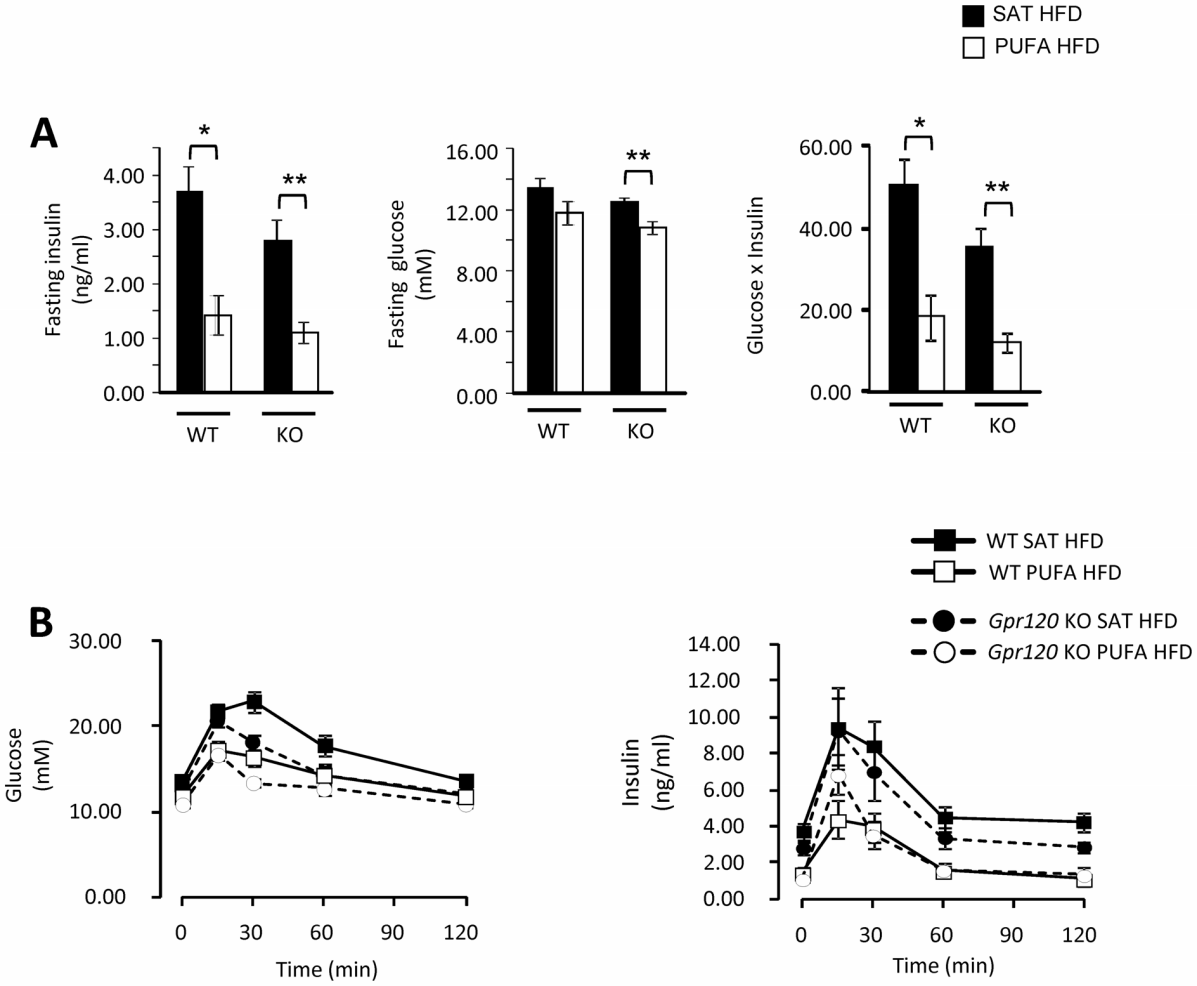
Oral glucose tolerance test. A; Fasting blood insulin, glucose levels and insulin resistance index (glucose (mM) x insulin (ng/ml)) and B; oral glucose tolerance test in WT mice fed SAT HFD (n = 8, filled bars, filled square) or PUFA HFD (n = 8, open bars, open square) and in *Gpr120* KO mice fed SAT HFD (n = 7, filled bars, dashed lines filled circle) or PUFA HFD (n = 7, open bars, dashed lines open circle). Statistical calculations were based on individual area under the curve (AUC) values, calculated from 0 to 120 minute time points. Statistical analysis was done by 1-way ANOVA followed by two comparisons (SAT HFD *vs*. PUFA HFD) using Student's t-test, * p<0.05; ** p<0.01.

Measurement of fasting plasma levels of glucose and insulin as well as oral glucose tolerance tests (OGTT) were performed 14 weeks after the HFDs were introduced. Both WT and *Gpr120* KO had significantly lower fasting insulin levels on PUFA HFD than on SAT HFD. In contrast, Gpr120 KO mice, but not WT mice, had significantly lower fasting plasma glucose levels on PUFA HFD as compared to SAT HFD. An insulin resistance index (glucose (mM) x insulin (ng/ml)) was calculated and it was significantly lower in both groups of mice on PUFA HFD than in those on SAT HFD (**Fig. 5A**). Oral glucose tolerance was improved in both WT and *Gpr120* KO mice fed PUFA HFD compared to SAT HFD (**Fig. 5B**). In WT mice, blood glucose area under the curve (AUC) was 1714.1±110.5 on PUFA HFD and 2151.4±103.5 on SAT HFD (p<0.05), and in *Gpr120* KO mice, blood glucose AUC was 1532.5±47.0 on PUFA HFD and 1817.1±50.6 on SAT HFD (p<0.01). The insulin response measured as AUC was significantly lower following the glucose challenge in both genotypes when fed the PUFA HFD as compared to the SAT HFD. In WT mice, blood insulin AUC was 257.6±53.4 on PUFA HFD and 683.5±107.6 on SAT HFD (p<0.01), and in *Gpr120* KO mice, blood insulin AUC was 304.6±50.6 on PUFA HFD and 554.0±84.7 on SAT HFD (p<0.05). The 15 minute insulin response in *Gpr120* KO mice on PUFA HFD was more marked and correlated with a trend towards lower blood glucose levels at 30 minutes in the *Gpr120* KO mice compared to WT mice on PUFA HFD (**Fig. 5B**).

### Tissue weights and histology

Final body weight was 18% lower in WT mice and 12% lower in *Gpr120* KO mice on PUFA HFD as compared to the corresponding groups on SAT HFD (**Table 2**). Interestingly, the relative weights of epididymal and retroperitoneal fat depots tended to be higher in WT animals and was significantly higher in *Gpr120* KO animals on PUFA HFD as compared to those on SAT HFD. However, there was no effect on diet or genotype on relative brown adipose tissue (BAT) weights. The relative liver weight was approximately 40% lower in both WT and *Gpr120* KO animals on PUFA HFD.

**Table 2:** Absolute and relative tissue weights.

*Parameter\Genotype*	WT (n = 8) SAT HFD	WT (n = 8) PUFA HFD	*Gpr120* KO (n = 7) SAT HFD	*Gpr120* KO (n = 7) PUFA HFD	1-way ANOVA
**Body weight (g)**	53.50±1.12	43.83±3.05*	50.03±1.20	43.90±2.08*	p<0.05
**Lung** (g)	0.17±0.00	0.18±0.01	0.16±0.00	0.18±0.01	NS
Rel. lung (mg/g bw)	3.11±0.04	4.31±0.29***	3.25±0.07	4.11±0.07***	p<0.05
**Heart** (g)	0.19±0.01	0.17±0.01	0.18±0.00	0.18±0.01	NS
Rel. Heart (mg/g bw)	3.58±0.11	4.03±0.17*	3.66±0.07	4.12±0.13**	p<0.05
**Epi WAT** (g)	1.69±0.14	1.91±0.23	2.07±0.12	2.27±0.14	NS
Rel. epi WAT (mg/g bw)	31.81±3.09	42.72±4.48	41.73±3.44	51.54±0.98*	p<0.05
**Retro WAT** (g)	0.59±0.03	0.55±0.07	0.62±0.04	0.70±0.03	NS
Rel. retroWAT (mg/g bw)	11.00±0.62	12.38±1.63	12.47±0.98	16.08±0.57**	p<0.05
**BAT (g)**	0.54±0.04	0.49±0.07	0.51±0.04	0.40±0.04	NS
Rel. BAT (mg/g bw)	10.08±0.67	10.76±1.14	10.23±0.62	8.95±0.65	NS
**Testis (g)**	0.22±0.00	0.22±0.01	0.22±0.01	0.22±0.01	NS
Rel. Testis (mg/g bw)	4.03±0.11	5.29±0.43**	4.35±0.17	5.11±0.27*	p<0.05
**Liver (g)**	4.33±0.34	2.19±0.22***	3.38±0.29	1.84±0.07***	p<0.05
Rel. liver (mg/g bw)	80.21±5.09	49.60±2.57***	67.13±4.62	42.20±1.02***	p<0.05
**Kidney (g)**	0.43±0.02	0.42±0.02	0.40±0.01	0.47±0.03	NS
Rel. Kidney (mg/g bw)	8.03±0.28	9.84±0.50**	8.08±0.13	10.75±0.38***	p<0.05

Values are presented as group mean ± SEM. Statistical analysis performed by 1-way ANOVA followed by Students T-test comparing SAT HFD *vs*. PUFA HFD Star indicates significant difference between mice fed SAT HFD vs. WT fed PUFA HFD.

* p<0.05; ** p<0.01;

*** p<0.001.

WAT; white adipose tissue, Epi; Epididymal, Retro; retroperitoneal, BAT; brown adipose tissue, bw; body weight.

**Figure 6:**
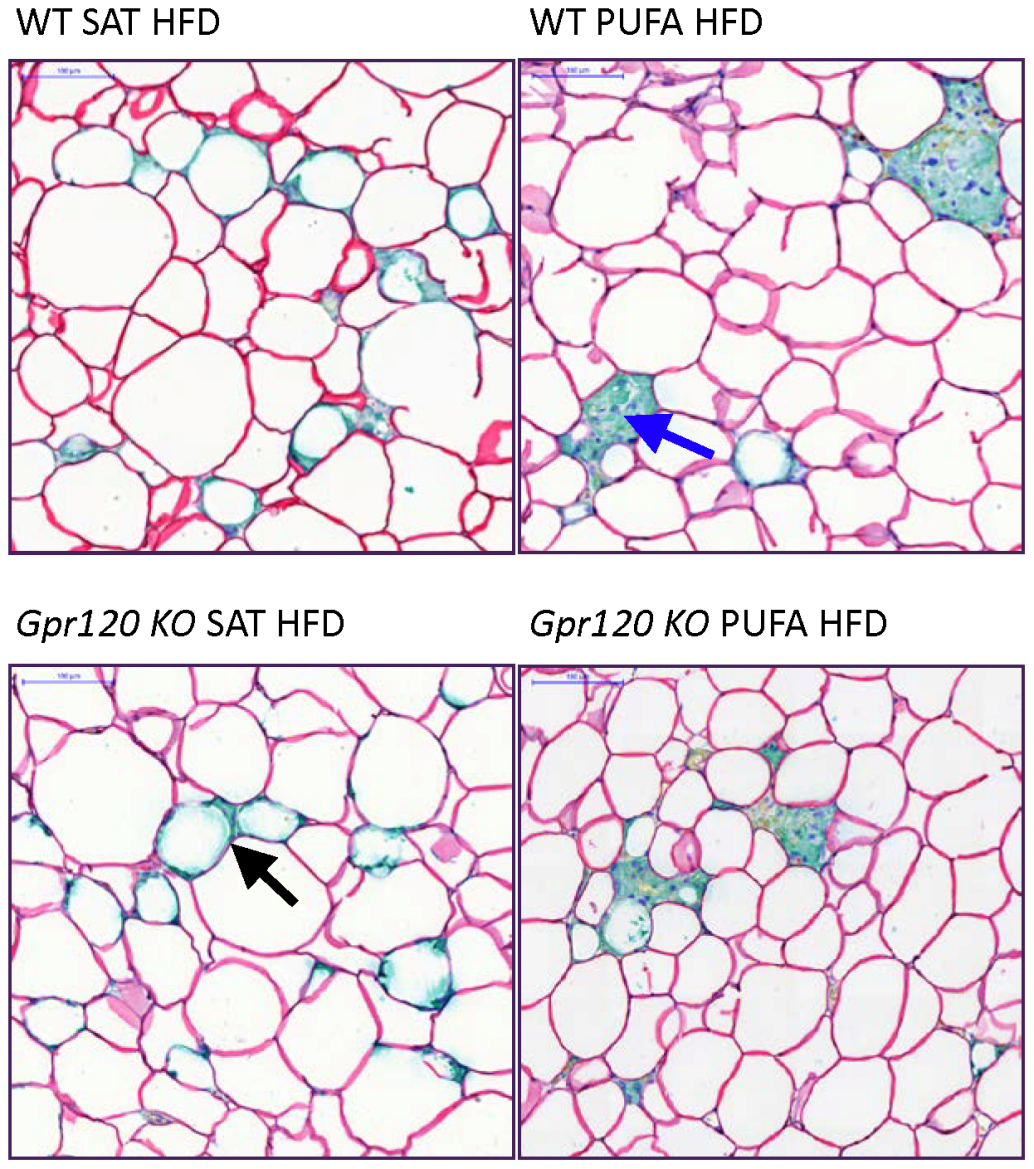
Adipose tissue histology. Representative slides of epididymal WAT double-stained for Perilipin and Mac2 (Macrophage 2 antigen, Galectin-3) from WT and *Gpr120* KO mice fed either the SAT HFD or PUFA HFD as indicated. Perilipin staining is seen as read coloured lines surrounding the cells. Some cells, typically associated with ‘crown like’ structures (CLS) do not display perilipin staining. Arrows indicate CLS (black arrow) and multinuclear giant cell aggregate (blue arrow). Scale bar upper left corner  = 100 µm.

Epididymal white adipose tissue (WAT) was analysed in terms of macrophage content. No significant differences in Mac2 quantified staining were observed between PUFA HFD and SAT HFD fed mice. In WT mice, Mac2 area was 1.14±0.23% on PUFA HFD and 0.98±0.34% on SAT HFD, and in *Gpr120* KO mice, the Mac2 area was 0.98±0.21% on PUFA HFD and 0.80±0.22% on SAT HFD (representative slides shown in **S3 Fig** **.**). WAT tissue was also double stained with Perilipin and Mac2 to understand how the different pattern of immune markers correlated with dead adipocytes (**Fig. 6**). As expected, adipose tissue from mice fed SAT HFD displayed high number of ‘crown like’ structures (CLS) surrounding perilipin-free lipid droplets (**Fig. 6** and **S3 Fig** **.**). Interestingly, staining of the WAT macrophages in mice fed the PUFA HFD revealed the presence of similar numbers of immunopositive macrophages but these displayed a different pattern of Mac2-staining as multinuclear giant cells aggregation (MNGCA) rather than CLS, and only a few of the CLS were observed in samples from the mice fed PUFA HFD (**Fig. 6**). Most cells on the slides displayed positive perilipin staining, regardless of genotype and diet. However, WAT tissue slides from mice fed SAT HFD displayed more frequent perilipin negative cells associated with the CLS compared to mice fed PUFA HFD. Slides from PUFA HFD fed mice had less perilipin free cells, indicating that PUFA HFD feeding is associated with fewer dead adipocytes compared to SAT HFD feeding. No differences between genotypes were observed with respect to Mac2 and perilipin staining.

**Figure 7:**
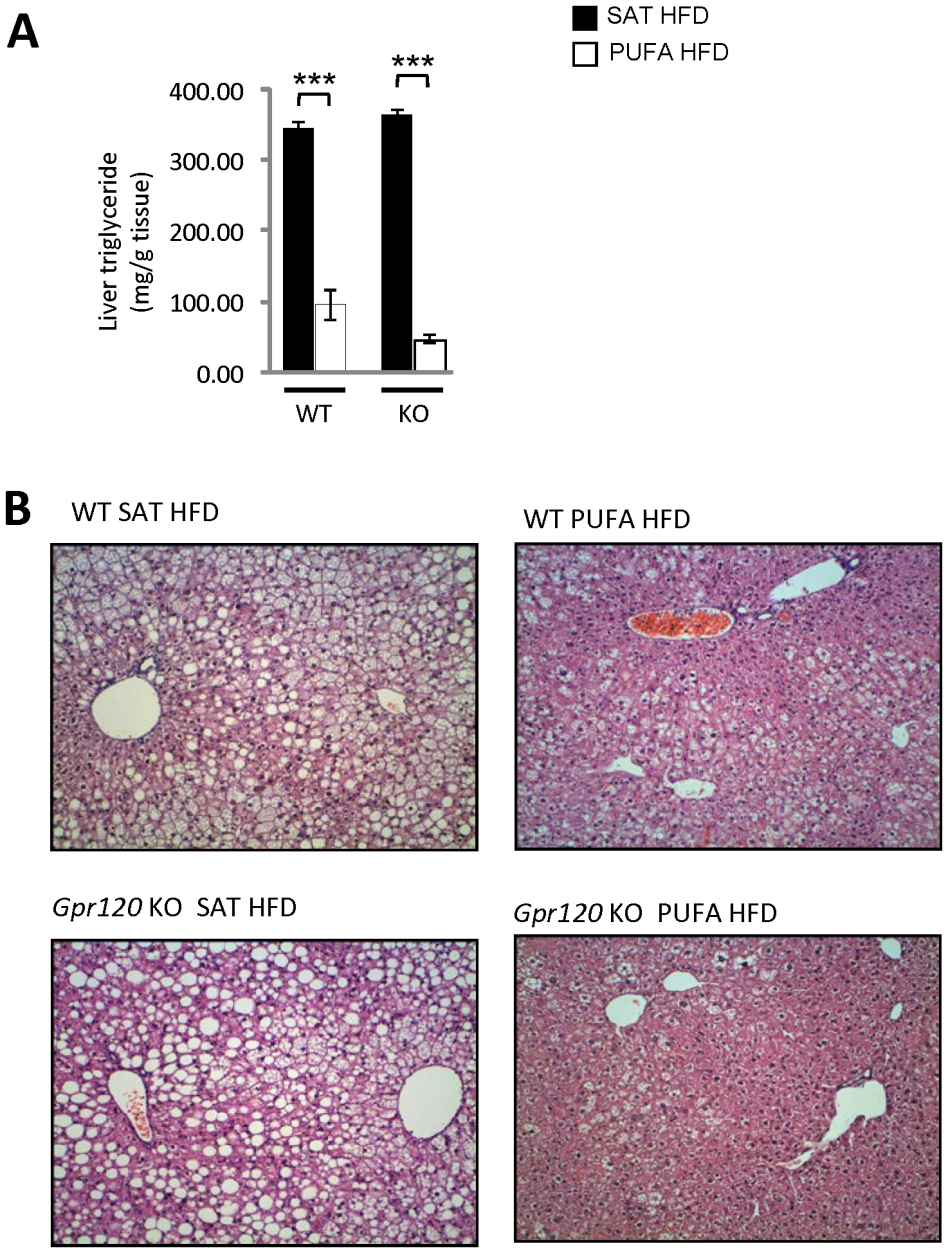
Liver triglyceride content and histological assessment. A; Hepatic triglyceride content was measured at 31 weeks of age after 18 weeks of HFD in WT mice fed SAT HFD (n = 8, filled bars) and PUFA HFD (n = 8, open bars) and in *Gpr120* KO mice fed SAT HFD (n = 7, filled bars) and PUFA HFD (n = 7, open bars). Statistical analysis was done by 1-way ANOVA followed by two comparisons (SAT HFD *vs*. PUFA HFD) using Student's t-test, *** p<0.001. B; Representative slides of livers stained by hematoxylin- eosin (H&E) from WT and *Gpr120* KO mice fed either the SAT HFD or the PUFA HFD as indicated.

The inferior lobes of the liver were sampled to assess liver triglyceride content and histopathology. WT mice fed PUFA HFD displayed about 70% lower hepatic triglyceride content compared to the SAT HFD fed WT mice. *Gpr120* KO mice fed PUFA HFD had close to 90% lower liver triglyceride content compared to *Gpr120* KO mice fed the SAT HFD (**Fig. 7A**). These findings were supported by histopathological examination, which revealed that the PUFA HFD fed mice, regardless of genotype, displayed a lower degree of hepatic steatosis compared to animals fed the SAT HFD. The steatosis was graded from 0 to 5 and mean steatosis grade was 3.9±0.1 in WT and 4.0±0.0 in *Gpr120* KO mice on SAT HFD. On PUFA HFD, the steatosis grade was 1.6±0.4 in WT animals and 0.6±0.3 in *Gpr120* KO mice. In addition, liver samples from PUFA HFD fed WT and *Gpr120* KO mice showed conspicuous sinusoidal Kupffer cells and/or possibly perisinusoidal Ito cells. These cells had a foamy appearance with markedly swollen and slightly basophilic cytoplasm, and they were sometimes surrounded by inflammatory cells (**Fig. 7B**).

**Figure 8:**
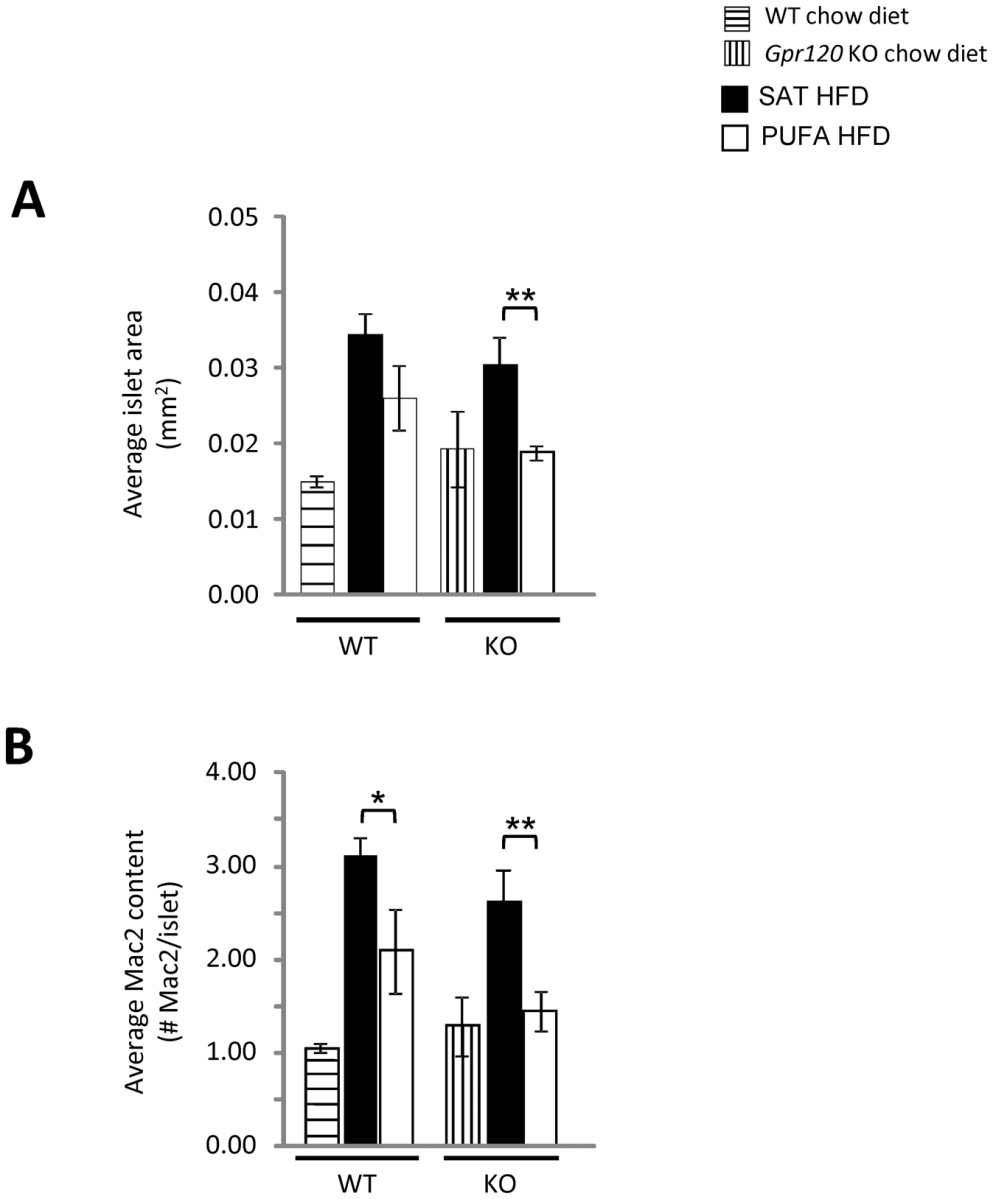
Pancreas histology. A; Average islet area and B; macrophage content in WT (n = 3, horizontally lined bars) and Gpr120 KO (n = 3, vertically lined bars) fed chow diet, WT mice fed SAT HFD (n = 8, filled bars) and PUFA HFD (n = 8, open bars) and in *Gpr120* KO mice fed SAT HFD (n = 7, filled bars) and PUFA HFD (n = 7, open bars). Statistical analysis was done by 1-way ANOVA followed by two comparisons using Student's t-test, SAT HFD *vs*. PUFA HFD, * p<0.05; ** p<0.01.

Pancreases were analyzed to determine the average islet area and macrophage content. Separate cohorts of chow fed WT and *Gpr120* KO mice were also included to understand islet size and inflammation under normal dietary conditions. No significant difference was observed in islet area between PUFA HFD fed and SAT HFD fed WT mice (**Fig. 8A**). However, the PUFA HFD fed WT mice displayed lower numbers of macrophages per islet compared to the SAT HFD fed mice (PUFA HFD: 2.09±0.45 cells/islet, SAT HFD: 3.11±0.19; p = 0.05). *Gpr120* KO mice fed PUFA HFD had significantly lower islet area and macrophage content per islet compared to *Gpr120* KO mice fed SAT HFD (**Fig. 8B**).

### Plasma analysis

Plasma levels of adiponectin were higher and ALAT levels were lower in PUFA HFD fed *vs*. SAT HFD fed WT and *Gpr120* KO mice (**Table 3**). Plasma leptin levels trended to be lower in PUFA HFD fed mice, but the effect was not statistically significant. Plasma levels of albumin were significantly lower in PUFA HFD fed vs. SAT HFD fed WT mice, but no significant effect of diet on plasma albumin was observed in *Gpr120* KO mice. Plasma cholesterol levels were significantly lower and there was a trend towards lower plasma triglyceride levels in the mice fed PUFA HFD compared to mice given SAT HFD. There was no effect of the diets on fructosamine levels. The fatty acid composition of triglycerides, phospholipids and cholesteryl esters were determined in plasma. The content of C20:5*n*-3was below the detection limit in the SAT HFD mice and PUFA HFD diet resulted in high levels of this fatty acid. The levels of 22:6*n*-3 and C18:3*n*-3 were significantly higher in mice fed PUFA HFD than in mice fed SAT HFD. The mol% of n-3 PUFA (22:6*n*-3, C20:5*n*-3, C18:3*n*-3) was 38.2% and 39.3% in WT and *Gpr120* KO mice fed PUFA HFD respectively, and 7.2 and 9.3% in WT and *Gpr120* KO mice fed SAT HFD respectively. Mice fed PUFA HFD, regardless of genotype had increased levels of C14:0 and C16:1, but significantly lower levels of C16:0, C18:0, C18:1*n*-9, C18:2*n*-6 and C20:4*n*-6 compared to mice fed the SAT HFD (**Table 3**).

**Table 3:** Plasma analyses.

*Parameter\Genotype*	WT (n = 8) SAT HFD	WT (n = 8) PUFA HFD	*Gpr120* KO (n = 7) SAT HFD	*Gpr120* KO (n = 7) PUFA HFD	1-way ANOVA
Total bilirubin (µM)	1.78±0.10	1.80±0.15	1.81±0.15	1.81±0.12	NS
Albumin (g/l)	32.50±0.54	29.58±0.26***	30.96±0.35	29.56±0.61	p<0.05
ALAT (µkat/l)	7.46±0.99	0.62±0.08***	4.75±0.59	0.50±0.04***	p<0.05
Adiponectin (nM)	147.64±18.65	275.21±18.31***	191.23±11.27	335.68±30.46***	p<0.05
Leptin (ng/ml)	68.30±4.41	63.43±1.92	64.54±3.04	52.36±5.17	p<0.05
Cholesterol (mM)	6.92±0.20	3.64±0.30***	6.16±0.25	2.90±0.16***	p<0.05
Triglycerides (mM)	0.96±0.15	0.64±0.15	0.69±0.08	0.55±0.08	NS
Fructosamine (µM)	72.49±2.75	68.85±5.54	70.46±3.69	67.30±2.37	NS
C20:5n-6 (µM)	<LLOQ	163.9±14.6	<LLOQ	137.3±6.4	-
C22:6n-3 (µM)	93.2±4.6	129.6±8.2**	76.5±6.5	110.9±5.5**	p<0.05
C14:0 (µM)	48.4±2.6	88.2±3.8***	62.3±2.4	114.8±7.5***	p<0.05
C16:0 (µM)	468.2±29.9	207.7±22.1***	362.5±34.6	165.3±7.6***	p<0.05
C16:1n-9 (µM)	20.3±1.6	31.8±3.9*	16.6±1.2	28.6±1.9***	p<0.05
C18:0 (µM)	282.7±10.2	119.1±9.5***	230.6±20.0	98.0±4.5***	p<0.05
C18:1n-9 (µM)	278.2±22.6	67.1±13.7***	213.4±21.6	50.4±4.4***	p<0.05
C18:2n-6 (µM)	271.8±15.6	22.9±2.1***	224.5±21.2	18.0±0.9***	p<0.05
C18:3n-3 (µM)	43.2±1.1	88.7±3.1***	63.2±5.1	109.1±4.1***	p<0.05
C20:4n-6 (µM)	394.8±17.3	91.6±3.9***	323.9±25.9	77.6±2.0***	p<0.05
% n-3 of total	7.2±0.2	38.2±0.8***	9.3±0.5	39.3±0.4***	p<0.05

Values are presented as group mean ± SEM. Statistical analysis performed by 1-way ANOVA followed by Students t-test comparing SAT HFD *vs*. PUFA HFD. Significance tests were made between mice fed SAT HFD *vs*. mice fed PUFA HFD.

* p<0.05;

** p<0.01;

*** p<0.001.

Percentage of *n-*3 fatty acids in plasma triglycerides, phospholipids and cholesteryl esters were calculated as sum of 18:3*n*-3, 20:5*n*-3 and 22:6*n*-3 divided by the sum of all detected fatty acids. LLOQ - Lower Limit Of Quantification.

## Discussion

The n-3 polyunsaturated fatty acids (n-3 PUFAs) are generally considered to be beneficial for a variety of indications, including various aspects of the metabolic syndrome such as dyslipidemia, insulin resistance, endothelial dysfunction and inflammation [15], [22]–[24]. Recently, GPR120 has been implicated in several processes associated with metabolic regulation and body weight control [2], [5], [6] [8] and, in particular, the receptor has been proposed to mediate the effects of n-3 PUFA on these processes [5]. Against this background, we have investigated the well-known effects of a diet enriched in n-3 PUFA as compared to a diet comprising mainly of saturated and monounsaturated fatty acids on glucose and energy metabolism in a newly developed *Gpr120* deficient (*Gpr120* KO) mouse line. We found that wild type (WT) and *Gpr120* KO mice developed a similar level of obesity and impaired glucose control when fed a saturated HFD containing low amounts of n-3 PUFAs. To our surprise, when fed the n-3 PUFA enriched HFD, *Gpr120* deficient mice displayed similar body weight, body fat mass, liver fat, energy metabolism and glucose control to WT mice on n-3 PUFA HFD, showing that GPR120 is dispensable for the positive effects of n-3 PUFA on metabolism.

The effects of n-3 PUFA supplementation on body weight regulation and glucose control in rodents are well studied. In accordance with expectation, consumption of a high fat diet (HFD) containing n-3 PUFA resulted in lower body weight gain and adipose tissue mass than feeding of a HFD containing mainly saturated fat in C57Bl/6 mice [25]–[27]. Similarly, *fat-1* transgenic mice that express a desaturase from *C. Elegans*, which converts n-6 PUFA to n-3 PUFA, also show lower body weight gain [28] as compared to WT mice fed HFD. Our study showed that 11 weeks of PUFA HFD feeding resulted in lower total body fat mass compared to a corresponding group fed SAT HFD. Surprisingly, the weights of the epididymal and retroperitoneal fat depots were not different between the diets at the time of sacrifice, although the total body weight was about 20% lower in the PUFA HFD fed mice. This finding is in line with increased epididymal fat pad weight in spite of reduced body weight gain in mice on menhaden oil diet in a previous study [27]. Based on our own data and previous studies showing reduced body fat mass following n-3 PUFA supplementation [25], [26], the most likely explanation for the discrepant effect on total body fat mass and unchanged weight of the fat pads is a change in body fat distribution, e.g. reduced subcutaneous and visceral fat mass.

Even though the present study was not designed to evaluate the effect of a SAT HFD as compared to chow fed mice, it was clearly demonstrated that *Gpr120* deficiency did not result in a different body weight on a SAT HFD as compared to WT mice. This finding is in contrast to the reported intolerance to HFD observed by Ichimura *et.al.* [6]. In contrast to our results, they found that *Gpr120* deficient mice given a HFD containing mainly saturated and monounsaturated lipids displayed higher body weight gain, impaired glucose control and hepatic steatosis by comparison to WT mice fed HFD [6]. Suckow *et.al.* [8] using the same strain of *Gpr120* deficient mice as Oh *et.al.* [5], but back-crossed to C57bl/6, showed that *Gpr120* KO mice on HFD were initially heavier than the WT controls, but after 4 months there were no difference in body weight between the genotypes. We have done another study (data not shown) in which we fed WT (n = 7) and *Gpr120* KO mice (n = 9) a HFD with 60% energy from fat (Product nr: 12492, Research Diets Inc.). The mice were on the diet for 25 weeks and body weight gain, body composition, indirect calorimetry, oral glucose tolerance and food intake were recorded. However, none of these parameters were significantly different between WT and *Gpr120 KO* mice using that diet. Hence, the *Gpr120* deficiency model used herein is not associated with an abnormal metabolic phenotype.

Since the effects of n-3 PUFA HFD on food intake and energy expenditure were similar between the genotypes, it is concluded that GPR120 is not obligatory for n-3 PUFA mediated effects on energy metabolism. In spite of increased food intake and energy uptake, also in relation to lean body mass, the PUFA HFD fed mice gained less body weight than the SAT HFD fed mice independent of genotype. Therefore, it is likely that increased energy expenditure explains the lower body weight gain during PUFA HFD as compared to feeding the SAT HFD. The energy expenditure is presented per individual mouse and also relative to lean body mass since body fat mass mainly explained the difference in body weight gain and energy expenditure of adipose tissue is considered to be relatively low [29]. Total energy expenditure per mouse was significantly lower in WT mice on PUFA HFD than in WT mice on SAT HFD, but no significant difference was observed between the *Gpr120* KO diet groups. The difference in total energy expenditure per mouse between WT mice given SAT HFD and WT mice given PUFA HFD could be explained by the lower body weight (see body weights at 10 weeks in Fig. 2) and similar body composition of the *Gpr120* KO mice on SAT HFD as compared to WT mice on SAT HFD. Surprisingly, there was no detectable difference in energy expenditure related to lean body mass in spite of higher food intake related to lean body mass and lower body weight gain in mice on PUFA HFD. The measurements of energy expenditure were performed at thermoneutrality to avoid the influence of heat loss and therefore heat production. However, all other experiments were performed at room temperature. It is possible that the difference in energy expenditure between the mice on different diets was too small to be detected by the system or that there had been a difference in energy expenditure if the oxygen consumption had been measured at room temperature. On the other hand, energy expenditure at thermoneutral temperature has been shown to be increased by n-3 PUFA supplementation in a rat model [30]. Several potential and non-exclusive mechanisms for increased energy expenditure following n-3 PUFA diet have been described, including increased sodium and calcium pump activities, increased mitochondrial proton leak and an enhanced futile cycle in adipocytes involving lipolysis and re-esterification [14], [31], [32]. Therefore, a third possibility is that PUFA HFD triggered increased energy expenditure mainly in the adipose tissue. Few studies have been performed in humans on the effects of n-3 PUFA on energy metabolism but the available evidence suggests that the effects are small and in line with previous results from rodent studies [14]. For example, a cross-over study in healthy volunteers showed that n-3 PUFA supplementation resulted in an increase in basal metabolic rate and reduced adipose tissue mass [33].

A HFD enriched in n-3 PUFA or transgenic over expression of *fat-1* have been shown to improve glucose control in mice, including fasting plasma glucose, glucose tolerance and several measures of insulin sensitivity [12], [13], [26], [27], [34], [35] reviewed in [14]. In human studies, n-3 PUFA supplementation often improves glucose control in non-diabetics but the results are less clear in type 2 diabetes [15]. Oh and co-workers showed that n-3 fatty acid supplementation for 5 weeks resulted in improved glucose metabolism by improving insulin sensitivity in WT but not in *Gpr120* deficient mice [5]. The importance of GPR120 in the regulation of insulin sensitivity was recently challenged [8]. Suckow *et.al*. showed that the *Gpr120* deficient mice have an enhanced glucagon secretion and sensitivity, which better explained the deteriorated glucose control than worse insulin resistance. Islet studies showed that *Gpr120* deficiency enhanced arginine stimulated glucagon secretion, while *Gpr120* deficiency reduced glucagon response to DHA and palmitic acid, which would indicate an improved glucose control in *Gpr120* KO mice on HFD [8]. In our study, the PUFA HFD had similar effects on glucose control in WT and *Gpr120* deficient mice. If anything, the *Gpr120* deficient mice on PUFA HFD displayed a healthier phenotype including significantly lower fasting glucose levels and a more marked insulin response at 15 minutes post glucose challenge as compared to the SAT HFD.

Adipose tissue histology showed similar number of macrophages following PUFA HFD as compared to SAT HFD. However, the distribution of macrophages was markedly different with less CLS and less perilipin-free lipid droplets in the adipose tissue of mice given the PUFA HFD as compared to mice given SAT HFD. However, we did not observe any difference between the genotypes in terms of CLS or presence of perilipin-free lipid droplets. The lower number of CLS following treatment with n-3 PUFA as compared to a diet enriched in saturated fatty acids is in line with previous studies [5], [12], [36]. In contrast to our findings, these studies also showed reduced number of adipose tissue macrophages as a consequence of increase in n-3 PUFA [5], [12], [36]. Instead of a reduced number of macrophages, we observed that n-3 PUFA treatment resulted in accumulation of macrophages as multinuclear giant cells aggregation (MNGCA). The mechanism responsible for the n-3 PUFA induced aggregation of macrophages into multinuclear giant cells instead of prevention of migration of macrophages into the adipose tissue is at the present unknown. In summary, the n-3 PUFA enriched diet showed reduced number of CLS and dead adipocytes, while no apparent difference between WT and *Gpr120* KO mice was observed.

We observed a markedly lower liver triglyceride content in mice on PUFA diet compared to the saturated/monounsaturated diet, independent of genotype. If anything, the liver lipid content was lower in the *Gpr120* deficient than in WT animals fed PUFA diet. This result is in sharp contrast to the finding that *Gpr120* deficient mice were refractory to the n-3 PUFA diet with respect to liver fat in another study [5]. We observed markedly higher plasma adiponectin levels in the mice given the PUFA-enriched diet, an effect in line with previous studies [26], [37]. Further, the effect was similar in WT and *Gpr120* deficient mice. Adiponectin is an important regulator of glucose homeostasis and liver fat content [38], [39], and therefore is a plausible mediator of the positive effects of n-3 PUFA on glucose- and lipid metabolism.

The Langerhans islets in mice fed PUFA HFD were smaller and contained fewer macrophages than those from mice fed the SAT HFD. This effect was, if anything, more pronounced in the *Gpr120* deficient mice. A number of factors might have contributed to this effect. First, body weight and total body fat was lower in the PUFA diet mice. Second, since glucose tolerance was improved in spite of reduced insulin response, the demand for insulin production and therefore that aspect of beta-cell stress was clearly reduced in mice given the PUFA diet as compared to the SAT HFD. Certainly, our results are consistent with the earlier work showing PUFA, or more specifically EPA, reduces the negative effects of long-chain saturated fatty acids on beta-cell function and survival [40]. Our results thus contrast with the study by Ichimura *et.al.* who reported that islets from *Gpr120* deficient mice were larger than those from WT mice on HFD, probably reflecting the worse insulin sensitivity in those mice [6]. In this study, we found no evidence for larger islet size in the *Gpr120* deficient mice than in WT animals, rather the opposite. Hence, the present results do not support negative effects of *Gpr120* deficiency on islet health *in vivo*.

An obvious question is the extent to which the present study protocol differs from other published protocols suggesting the importance of GPR120 for glucose and energy metabolism and whether this might explain the differing results [5], [6] [8]. Oh *et.al.* switched from a 60 energy% saturated HFD to a 27% menhaden oil replacement of the HFD during 5 weeks resulting in 25 mol% EPA and DHA in plasma lipids whereas in our study the mice were given the n-3 PUFA enriched diet for 18 weeks and the diet resulted in 38–39 mol% EPA, DHA and ALA (C18:3n-3) of total fatty acids in plasma lipids. Thus, in our work, the menhaden diet was given in larger quantities during a longer period of time which might have resulted in a larger effect on body weight gain. It is well know that reduced body weight gain and increased energy expenditure will improve metabolic impairments. Hence, it is conceivable that the effect on body weight gain in PUFA HFD fed animals could have obscured other, subtle, effects on glucose control which occur as a result of *Gpr120* deficiency. Two of the previously published *Gpr120* KO mouse lines were developed on a mixed 129SV and C57BL/6 genetic background and it is not clear if and to what extent these lines were bred towards one genetic background [5], [6]. However, a recent study used mice from Taconic backcrossed onto C57Bl/6 for 6 generations [8]. The *Gpr120* KO mouse line studied in this paper had a pure C57BL/6N genetic background and it is well established that the genetic background will affect the phenotype of experimental mice [41], [42]. Another technical factor is whether the DNA selection marker used to identify positive ES-cells is maintained or removed in the mice, as it is known that selection markers can influence phenotypes [43]. In the present study, the selection marker was removed from the *Gpr120* KO mouse line by a Cre-LoxP breeding program. A third possible difference between the *Gpr120* null mouse lines is the targeting strategy. The mice used by Oh *et.al.* and Suckow *et.al.* disrupted exon 2, whereas the line studied by Ichimura *et.al* and ourselves have targeted parts of exon 1. We ensured that no *Gpr120* transcript was present in the *Gpr120* deficient animals, either from exon 1 or from downstream exons, by designing the RT-PCR primers to amplify over the deleted DNA region as well as over exons 2–3. Moreover, the inhibition of glucose stimulated somatostatin secretion by a *Gpr120* agonist occurred in WT animals, but was lost in *Gpr120* KO animals [7], indicating lack of functional *Gpr120* expression in our Gpr120 deficient model. Finally, the X-gal staining showed the expected tissue distribution as compared to mRNA expression of the receptor [2], [4]–[6] [1].

In summary, the present study shows that the major effects of n-3 PUFA diet on energy, lipid and energy metabolism, including any increases in plasma adiponectin levels, are not mediated by GPR120. However, we cannot exclude the possibility that there may be less pronounced effects of n-3 PUFA mediated by the GPR120 receptor that were not revealed in this study because of the marked effect of n-3 PUFA on energy metabolism.

## Supporting Information

S1 Fig **(A)** ***Gpr120*** **gene targeting strategy.** Schematic diagram over the native 5′ region of *Gpr120* gene, targeting vector, targeted allele and the disrupted *Gpr120* gene. A region of 0.567 kb of the *Gpr120* exon 1 CDS was replaced in frame with a nuclear βGal expression cassette followed a loxP floxed PGK neo selection marker. Filled rectangles indicate 5′ un-translated region (UTR), horizontal bar indicates probe used for southern blotting and triangles indicate loxP sites. (B) Southern blot analysis of the targeted ES clones. Genomic DNA was digested with SexAI or SspI and probed with a probe shown in (A). Expected sizes of DNA fragments of the targeted allele are indicated in (A). Lane 1-6 represent targeted clones, lane 7 represent 1 kb marker.
(TIF)

S2 Fig **Indirect calorimetry assessment.** Energy expenditure assessed in kilocalories per hour per mouse (kcal/h) is shown in panel A for WT fed SAT HFD (n = 8, filled square) and PUFA HFD (n = 8, open square), and in panel B for *Gpr120* KO mice fed SAT HFD (n = 7, filled circle) and PUFA HFD (n = 7, open circle). Energy expenditure relative to lean body mass (LBM) is shown in panel C for WT fed SAT HFD (n = 8, filled square) and PUFA HFD (n = 8, open square) and in panel D for *Gpr120* KO mice fed SAT HFD (n = 7, filled circle) and PUFA HFD (n = 7, open circle). Thick black lines at the X-axis represent light off.
(TIF)

S3 Fig **Adipose tissue histology.** Representative slides of epididymal WAT stained for Mac2 (Macrophage 2 antigen, Galectin-3) from WT and *Gpr120* KO mice fed either the SAT HFD or the PUFA HFD as indicated.
(TIF)

S1 Table **Details of diet compositions and degree of lipid saturations in the PUFA and SAT HFD's.**
(DOCX)

S1 Supplementary experimental procedures Outlining details in experimental procedures
(DOCX)
