# Supplementary figures and images for: The Beneficial Effects of n-3 Polyunsaturated Fatty Acids on Diet Induced Obesity and Impaired Glucose Control Do Not Require Gpr120
Source: PLoS One. 2014 Dec 26;9(12):e114942. doi: 10.1371/journal.pone.0114942 (PMC4277291; doi:10.1371/journal.pone.0114942)

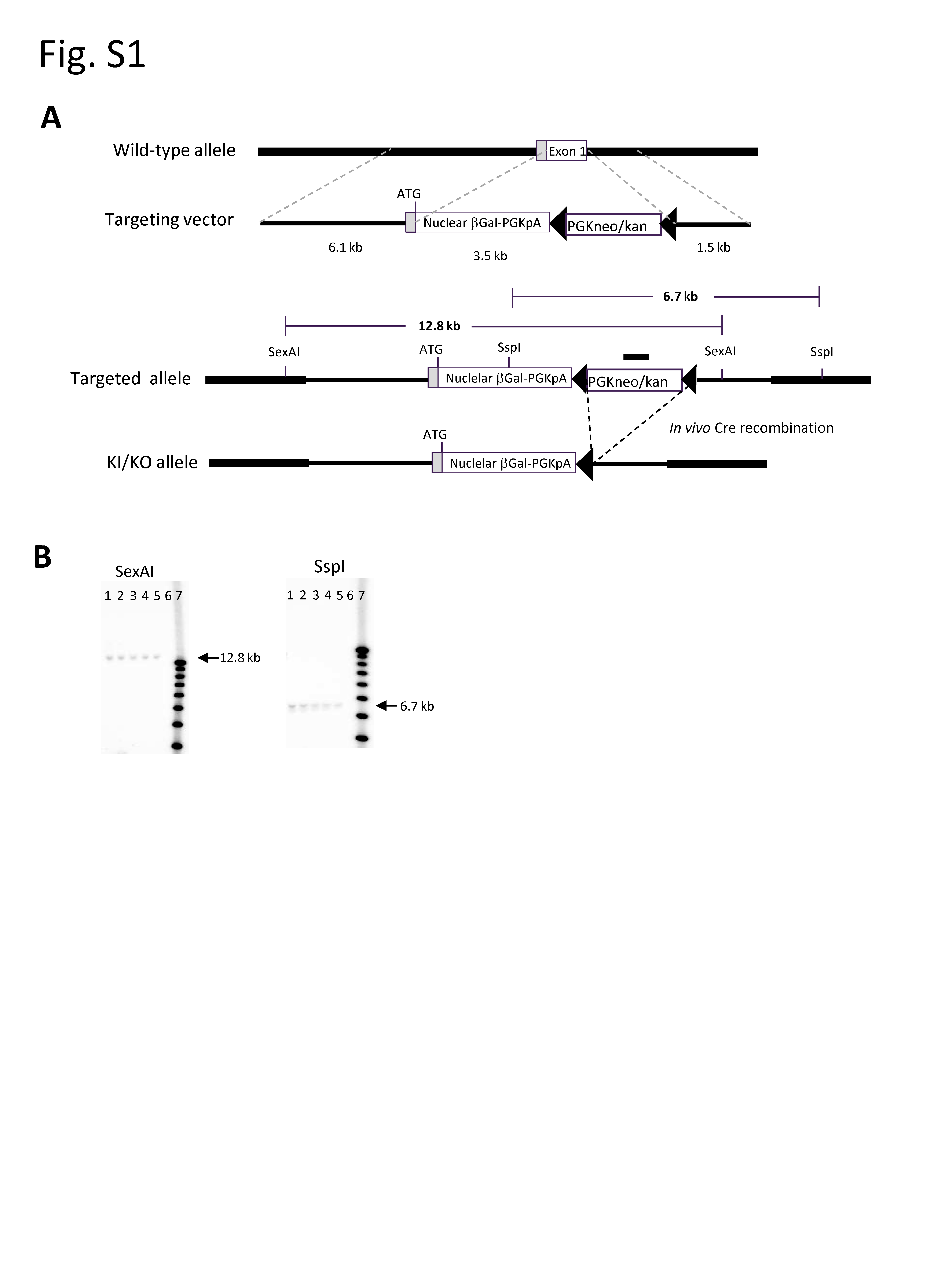

Supplement: S1 Fig — (A) Gpr120 gene targeting strategy. Schematic diagram over the native 5′ region of Gpr120 gene, targeting vector, targeted allele and the disrupted Gpr120 gene. A region of 0.567 kb of the Gpr120 exon 1 CDS was replaced in frame with a nuclear βGal expression cassette followed a loxP floxed PGK neo selection marker. Filled rectangles indicate 5′ un-translated region (UTR), horizontal bar indicates probe used for southern blotting and triangles indicate loxP sites. (B) Southern blot analysis of the targeted ES clones. Genomic DNA was digested with SexAI or SspI and probed with a probe shown in (A). Expected sizes of DNA fragments of the targeted allele are indicated in (A). Lane 1-6 represent targeted clones, lane 7 represent 1 kb marker. (TIF) [file pone.0114942.s001.tif]

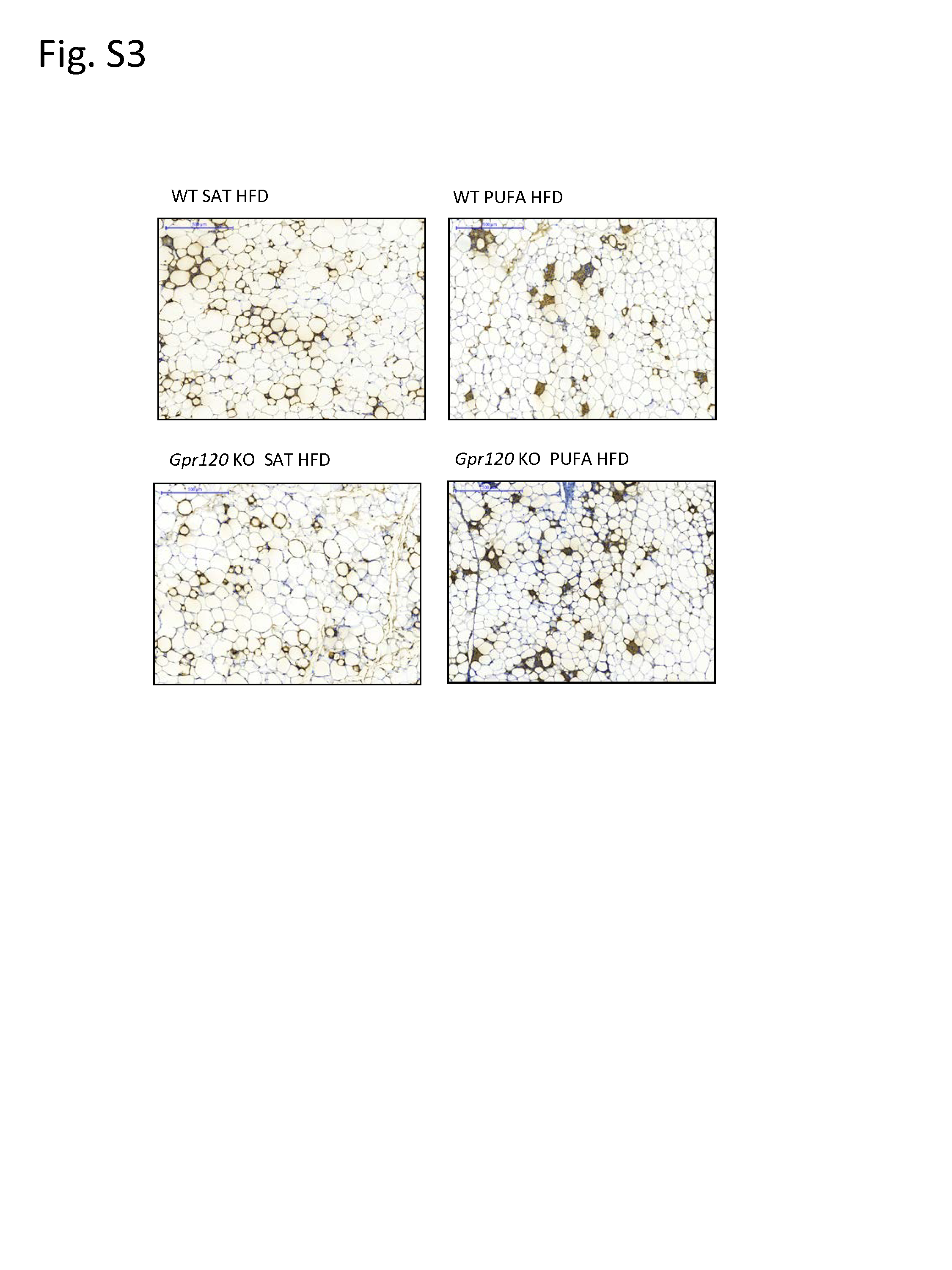

Supplement: S3 Fig — Adipose tissue histology. Representative slides of epididymal WAT stained for Mac2 (Macrophage 2 antigen, Galectin-3) from WT and Gpr120 KO mice fed either the SAT HFD or the PUFA HFD as indicated. (TIF) [file pone.0114942.s003.tif]
