# Supplementary material for: The Beneficial Effects of n-3 Polyunsaturated Fatty Acids on Diet Induced Obesity and Impaired Glucose Control Do Not Require Gpr120
Source: PLoS One. 2014 Dec 26;9(12):e114942. doi: 10.1371/journal.pone.0114942 (PMC4277291; doi:10.1371/journal.pone.0114942)
Supplement: S1 Table — Details of diet compositions and degree of lipid saturations in the PUFA and SAT HFD's. (DOCX) [file pone.0114942.s004.docx]

**Table S1**

| Diet/content | SAT HFD | | PUFA HFD | |
| --- | --- | --- | --- | --- |
|  | g | kcal | g | kcal |
| Protein | 23.7 | 20.0 | 23.7 | 20.0 |
| Carbohydrate | 41.4 | 35.0 | 41.4 | 35.0 |
| Fat | 23.6 | 45.0 | 23.6 | 45.0 |
| Kcal/g | 4.73 |  | 4.73 |  |
| Casein | 200.0 | 800.0 | 200.0 | 800.0 |
| L-Cystine | 3.0 | 12.0 | 3.0 | 12.0 |
| Corn Starch | 72.8 | 291.0 | 72.8 | 291.0 |
| Maltodextrin 10 | 100.0 | 400.0 | 100.0 | 400.0 |
| Sucrose | 172.8 | 691.0 | 172.8 | 691.0 |
| Cellulose | 50.0 | 0.0 | 50.0 | 0.0 |
| Lard | 101.3 | 911.0 | 0.0 | 0.0 |
| Palm oil | 101.3 | 911.0 | 0.0 | 0.0 |
| Menhaden oil | 0.0 | 0.0 | 202.5 | 1823.0 |
| Saturated FA (%) | 42.1 |  | 29.3 |  |
| Monounsaturated FA (%) | 45.2 |  | 24.0 |  |
| Polyunsaturated FA (%) | 12.7 |  | 46.6 |  |
| n-6 (g/kg) | 27.1 |  | 10.9 |  |
| n-3 (g/kg) | 1.8 |  | 75.2 |  |
| n-6/n-3 ratio | 15.33 |  | 0.14 |  |
