# Supplementary material for: The Beneficial Effects of n-3 Polyunsaturated Fatty Acids on Diet Induced Obesity and Impaired Glucose Control Do Not Require Gpr120
Source: PLoS One. 2014 Dec 26;9(12):e114942. doi: 10.1371/journal.pone.0114942 (PMC4277291; doi:10.1371/journal.pone.0114942)
Supplement: S1 Supplementary experimental procedures — Outlining details in experimental procedures (DOCX) [file pone.0114942.s005.docx]

**Supplementary experimental procedures**

**Generation of the Gpr120 null mice**

Fragments of 6.1 kb and 1.5 kb of genomic DNA from up- and downstream of exon 1 were prepared from a BAC clone (Invitrogen, Life Technologies, Stockholm, Sweden) and used for 5’ and 3’ homology, respectively. To create a seamless in frame fusion between 5’UTR of *Gpr120* and CDS of the *nβGal* gene, an adaptor was made by annealing two complementary oligos which contain part the of 3’ region of 5’UTR of the *Gpr120* gene with an endogenous *AfeI* site and a part of the 5’ region of *nβGal* CDS with an endogenous AgeI site, having the following sequence: forward 5’CAGATGAGCGCTCTCTCAGACAGCGGCGGGCGGCCGGGCGCCCGGCATGACTGCTCCAAAGAAGAAGCGTAAGGTACCGGTGGGTGA3’ and reverse 5’TCACCCACCGGTACCTTACGCTTCTTCTTTGGAGCAGTCATGCCGGGCGCCCGGCCGCCCGCCGCTGTCTGAGAGAGCGCTCATCTG3’. The annealed fragment was subsequently digested with *AfeI* and *AgeI* to generate blunt- and sticky-ended termini. The remainder of the *nβGal* CDS fragment was derived from an in house *nβGal* expression plasmid using *AgeI* and *NotI*. The gene targeting vector was constructed using standard cloning techniques. A fragment of the 3’ homology (*AfeI/KpnI*) region was first cloned into a modified pBlueScript plasmid, pBSK-pA-NeoKan, which contains a mouse PGK polyA signal sequence followed by a loxP floxed PGKneo-kan selection cassette. Fragments of the 5’ homology region (*SacII/AfeI*), the adaptor (*AfeI/AgeI*) and the *nβGal* CDS (*AgeI/NotI*) were cloned into the plasmid simultaneously by multiple fragments ligation. The final *Gpr120* gene targeting vector contained a 6.1 kb 5’ homology domain and an in frame fused *Gpr120* - *nβGal* CDS - polyA, a PGKneo selection cassette with a 1.5 kb 3’ homology region (**Supplementary figure 1A**). The in frame fusion of *Gpr120* and *nβGal* genes was confirmed by direct sequencing of the relevant regions of the targeting vector. The targeting vector was linearized and electroporated into C57Bl/6N mouse embryonic stem (ES) cells which were then grown in media containing G418 (200 μg/ml). Thus, the *Gpr120* deficient mouse line was established on a pure C57bl/6 genetic background. The candidate ES cell clones were screened by PCR and the gene targeting event was confirmed by southern blotting (**Supplementary figure 1B**). The targeted ES cells were injected into BALB/c blastocysts and implanted into the uterus of pseudo pregnant recipient female mice. Chimeric offspring were bred to C57Bl/6N mice (Charles River, Sulzfeld, Germany) to produce black coated heterozygous offspring, which were subsequently bred to *Rosa26Cre* transgenic mice (having pure C57Bl/6 genetic background) to delete the loxP floxed PGKneo cassette. This resulted in *Gpr120* deficient mice having *nβGal* knock-in at the relevant locus.

**Plasma lipid extraction**

Plasma lipids were extracted by two phase liquid/liquid extraction by methanol/butanol with heptadecanoic acid methyl ester added as internal standard. The extracts were evaporated under nitrogen stream. Fatty acid esters, such as triglycerides, cholesterol esters and phospholipids, but not free fatty acids, were transesterified into fatty acid methyl esters (FAMEs), by 4% sodium methoxide in anhydrous methanol. The FAMEs were extracted by water and heptane additions and the upper heptane phase were transferred to a new vial. Solvents were removed by evaporation under nitrogen and FAMEs were re-dissolved in heptane for GC analysis. The samples were injected on a HP6890 gas chromatograph (Agilent technologies, Santa Clara, CA, USA) equipped with a DB23 column (JW scientific, Folsom, CA, USA) and a flame ionization detector (FID).

**Pancreas histology**

Slides were de-waxed in Histoclear (2x5minutes) and then rehydrated for successive 1 minute incubation periods in 100% ethanol, 90% ethanol, 70% ethanol, 50% methanol and finally for 5 minutes in distilled water. Antigen retrieval was used to detect Mac2 immunopositivity and was achieved by incubation in 1mmol/l citrate buffer, pH6 in a pressure cooker in a microwave oven (800W) for 20 minutes, followed by 20 minutes of cooling at room temperature. Positive immunoreactivity was visualized using the Dako REAL Envision Detection System (Dako,Ely, UK) using 3,3′-diaminobenzidine (DAB) as chromogen. The sections were counterstained with haematoxylin and then dehydrated by successive 5 minute incubations in 50% ethanol, 70% ethanol, 90% ethanol, 100% ethanol (2x5 minutes) and histoclear (2x5 minutes). The sections were mounted in DPX mountant (xylene and dibutyl phthalate).

**WAT Histology**

Fresh WAT depots were fixed in 4% paraformaldehyde, dehydrated, embedded in paraffin, sectioned at 5 μm, deparaffinized, and rehydrated on slides. Immunohistochemistry of macrophages (Mac2/gelectin3) and adipocytes (Perilipin) was performed. Briefly, tissue sections were subjected to microwave antigen retrieval in 10 mM citrate buffer (pH 6.0) following IHC staining in autostainer (IntelliPath; Biocare, USA). The primary rat anti-mouse antibody Mac2/galectin3 (1:10,000, cl. M3/38; Cedarlane Laboratories) and Rabbit anti-mouse mAb to Perilipin (#9349, 1:200, Cell Signaling) were used and detected with BioCare MACH 3 AP kit (M3R533) and BioCare Rat on Mouse HRP kit (RT517), followed by counter stain in Hematoxylin. Slides were dehydrated and mounted with Pertex (HistoLab) or air dried and mounted with Mountquick Aquerous solution (Daido Sangyo). Later, the slides were scanned using the Mirax slide scanner (Carl Zeiss) and Mac2/Galectin3 staining was quantified using the BIOPIX software (BioPix, Gothenburg, Sweden).
